# Population pharmacodynamic model of bicarbonate response to acetazolamide in mechanically ventilated chronic obstructive pulmonary disease patients

**DOI:** 10.1186/cc10448

**Published:** 2011-09-14

**Authors:** Nicholas Heming, Christophe Faisy, Saïk Urien

**Affiliations:** 1 Medical Intensive Care Unit, European Georges Pompidou Hospital (AP-HP), Université Paris Descartes, Sorbonne Paris Cité, 20 rue Leblanc, 75908 Paris, France; 2 CIC-0109 Cochin-Necker Inserm, Unité de Recherche Clinique, Tarnier Hospital, (AP-HP) and E.A. 3620 Université Paris Descartes, Sorbonne Paris Cité, 27 Rue du Faubourg Saint-Jacques 75014 Paris, France

**Keywords:** COPD, mechanical ventilation, weaning, metabolic alkalosis

## Abstract

**Introduction:**

Acetazolamide is commonly given to chronic obstructive pulmonary disease (COPD) patients with metabolic alkalosis. Little is known of the pharmacodynamics of acetazolamide in the critically ill. We undertook the pharmacodynamic modeling of bicarbonate response to acetazolamide in COPD patients under mechanical ventilation.

**Methods:**

This observational, retrospective study included 68 invasively ventilated COPD patients who received one or multiple doses of 250 or 500 mg of acetazolamide during the weaning period. Among the 68 investigated patients, 207 time-serum bicarbonate observations were available for analysis. Population pharmacodynamics was modeled using a nonlinear mixedeffect model. The main covariates of interest were baseline demographic data, Simplified Acute Physiology Score II (SAPS II) at ICU admission, cause of respiratory failure, co-prescription of drugs interfering with the acid-base equilibrium, and serum concentrations of protein, creatinin, potassium and chloride. The effect of acetazolamide on serum bicarbonate levels at different doses and in different clinical conditions was subsequently simulated *in silico*.

**Results:**

The main covariates interacting with acetazolamide pharmacodynamics were SAPS II at ICU admission (*P *= 0.01), serum chloride (*P *< 0.001) and concomitant administration of corticosteroids (*P *= 0.02). Co-administration of furosemide significantly decreased bicarbonate elimination. Acetazolamide induced a decrease in serum bicarbonate with a dose-response relationship. The amount of acetazolamide inducing 50% of the putative maximum effect was 117 ± 21 mg. According to our model, an acetazolamide dosage > 500 mg twice daily is required to reduce serum bicarbonate concentrations > 5 mmol/L in the presence of high serum chloride levels or coadministration of systemic corticosteroids or furosemide.

**Conclusions:**

This study identified several covariates that influenced acetazolamide pharmacodynamics and could allow a better individualization of acetazolamide dosing when treating COPD patients with metabolic alkalosis.

## Introduction

Chronic obstructive pulmonary disease (COPD) is a leading cause of morbidity and mortality [1]. The natural history of the disease is marked by exacerbations that affect the prognosis of patients with COPD [2]. Noninvasive mechanical ventilation in the clinical setting of COPD exacerbations reduces mortality [3]. However, initiation of invasive mechanical ventilation may be necessary, for instance, after the failure of an initial trial of noninvasive mechanical ventilation. Weaning from mechanical ventilation can be particularly difficult and prolonged in COPD patients with severe preexisting airflow limitation [4].

Metabolic alkalosis is an acid-base disorder that occurs frequently in the critically ill [5]. Metabolic alkalosis leads to hypoventilation, which may cause difficulty in the weaning process. Metabolic alkalosis is characterized by an elevated serum pH level secondary to increased plasma bicarbonate (HCO_3_^-^) retention. Correction of this acid-base disorder increases both minute ventilation and partial pressure of oxygen (PaO_2_), potentially allowing COPD patients to be weaned more rapidly from mechanical ventilation [6,7]. Acetazolamide (ACET), when administered after proper fluid loading and potassium supplementation, is one of the most common drugs used to treat metabolic alkalosis. ACET decreases proximal tubular HCO_3_^- ^reabsorption through carbonic anhydrase (CA) inhibition in the luminal borders of renal proximal tubule cells [8]. In addition, ACET (1) induces CO_2 _retention by inhibiting CA isoenzymes in red cells and tissue and (2) improves cardiac function and gas exchange in cor pulmonale by stimulating diuresis [8].

There is a paucity of literature to support only one ACET dosing strategy during the weaning period for mechanically ventilated COPD patients. It has been established that a single 500-mg daily dose of ACET reverses metabolic alkalosis over 72 hours in intubated patients with COPD or asthma as effectively as multiple doses of 250 mg [9]. However, a recent study has shown that ACET administration at a daily dose of 500 mg during the weaning period of COPD patients with mixed or pure metabolic alkalosis only moderately diminishes serum HCO_3_^- ^levels, without changing either partial pressure of carbon dioxide (PaCO_2_) levels or minute ventilation [10]. The lack of effectiveness of ACET on respiratory mechanics could be due to alterations in ACET pharmacodynamics [10]. Indeed, ACET pharmacodynamics in ICU patients has received little attention, especially in mechanically ventilated COPD patients.

The objectives of our present study were to develop a population pharmacodynamic model for ACET in mechanically ventilated COPD patients using nonlinear mixed-effects modeling and to examine the possible effect of covariates on a pharmacodynamic parameter, the observed serum HCO_3_^- ^level.

## Materials and methods

Data for COPD patients admitted to the medical ICU of a tertiary care teaching hospital with the diagnosis of acute respiratory failure were retrospectively examined over a 10-year period (2000 to 2010). In accordance with French law, no local institutional review board authorization was needed, owing to the study's observational design, and the Commission Nationale de l'Informatique et des Libertés approved the use of computerized medical data with protection of patient confidentiality. COPD patients intubated for acute respiratory failure who had received ACET during the weaning period were eligible for analysis. COPD was diagnosed according to the Global Initiative for Chronic Obstructive Lung Disease criteria [11]. The weaning period was defined as the time between readiness to wean and extubation. Readiness to wean was defined according to the criteria of the Sixth International Conference Consensus in Intensive Care Medicine held in 2005 on the subject of weaning from mechanical ventilation [12]. Five hundred and thirty-six files of COPD patients admitted to our ICU were examined.

### ACET administration

Acetazolamide (Diamox; Sanofi-Aventis, Paris, France) administration (250 to 500 mg delivered two times per day via nasogastric tube or intravenously) was monitored by physicians in charge of the patient according to arterial blood gas analyses routinely performed. ACET was not administered to patients with liver or kidney failure.

### Patient data

Baseline demographic data at the time of ICU admission included patient age, sex, body weight, smoking history, use of long-term systemic corticosteroids or diuretics (including ACET), home oxygen therapy or home noninvasive ventilation, left ventricular ejection fraction assessed by echocardiography and prior pulmonary function tests performed while the patient was in stable condition, the Simplified Acute Physiology Score II (SAPS II) [13] and the cause of respiratory failure. Serum HCO_3_^- ^level was obtained by arterial blood gas analysis (ABL series; Radiometer Copenhagen, Copenhagen, Denmark) before and up to 24 hours after ACET administration. Serum levels of protein, creatinine, potassium and chloride were recorded during the three hours preceding ACET administration and up to 24 hours afterward. Throughout ACET treatment, data regarding the mode of mechanical ventilation and additional treatments which could alter acid-base equilibrium (furosemide, corticosteroids, β_2_-agonists and fluid loading) were collected from charts prospectively completed by physicians and nurses. We also collected length of invasive mechanical ventilation, length of ICU stay and ICU outcome. Among the parameters appraised, serum HCO_3_^- ^concentration was used in this pharmacodynamic analysis for direct relationship with ACET dosage.

### Weaning from mechanical ventilation

The weaning process was standardized by implementing a written protocol according to the recommendations of the Consensus Conference of the Société de Réanimation de Langue Française held in October 2001. The weaning strategy (1) consisted of a progressive decrease in pressure support ventilation or volume-assisted ventilation with the use of progressively increased time on a T-piece and (2) was chosen by physicians in charge according to the difficulty of the weaning process [12]. External positive end-expiratory pressure (PEEP) was applied to counterbalance intrinsic PEEP, typically 4 to 6 cmH_2_O, in COPD patients. During the weaning process, β_2_-agonists or furosemide was administered by attending physicians as indicated. In our ICU, the criteria for extubation were also standardized in a written protocol detailed elsewhere [10].

### Pharmacodynamic modeling

The pharmacokinetics of ACET were ascribed to a one-compartment open model with linear elimination [14,15]. Elimination half-life was fixed at six hours (0.25 hours/day) [14]. The corresponding differential equations used were for the intravenous or enteral route, respectively:

$$\text{dA}\left(\text{t}\right)∕\text{dt}=-k_{\text{elim}}\times \text{A}\left(\text{t}\right)$$

or

dG(t)∕dt=-ka×G(t),withdA(t)∕dt=ka×G(t)-ke lim×A(t),

where A(t) and *k*_elim_, respectively, denote the ACET amount in the body at a given time and the constant rate of elimination (half-life = 0.693/*k*_elim_), and G(t) and *k*_a _represent the ACET amount in the gut at a given time and the first-order absorption rate, respectively. Serum HCO_3_^- ^concentration (Bicar(t), in mmol/L) was ascribed to a turnover model as follows:

$$\text{dBicar}\left(\text{t}\right)∕\text{dt}=k_{\text{in}}-k_{\text{out}}\times \text{Bicar}\left(\text{t}\right),$$

where *k*_in _(mmol/L/day) and *k*_out _(day^-1^) are the HCO_3_^- ^rate formation and the first-order constant rate of HCO_3_^- ^elimination, respectively. At equilibrium, dBicar/dt = 0 and the HCO_3_^- ^baseline level was Bicar_0 _= *k*_in_/*k*_out_. The effect of ACET (E_ACET_) on HCO_3_^- ^equilibrium was assumed to increase the elimination process:

$$\text{dBicar}\left(\text{t}\right)∕\text{dt}=k_{\text{in}}-{\text{E}}_{\text{ACET}}\;\times k_{\text{out}}\times \text{Bicar}\left(\text{t}\right),$$

$$\text{with}\;{\text{E}}_{\text{ACET}}=1+{\text{E}}_{\max}\times \text{A}\left(\text{t}\right)∕\left[\text{A}\left(\text{t}\right)+{\text{A}}_{50}\right],$$

where A_50 _is ACET dosage that induces 50% of the maximal effect (E_max_).

### Data analysis

Data was analyzed using the nonlinear mixed-effect modeling software program Monolix version 3.1S Release 2 http://wfn.software.monolix.org/. The main covariates of interest were baseline demographic data, SAPS II at ICU admission, cause of respiratory failure, coprescription of drugs interfering with the acid-base equilibrium and serum concentrations of protein, creatinine, potassium and chloride. Parameters were estimated by computing the maximum likelihood estimation of the parameters without any approximation of the model (that is, no linearization) using the stochastic approximation expectation maximization algorithm combined with a Markov chain Monte Carlo procedure. A proportional error model was used to describe residual variability (ε_PROP _and ε_ADD_), and between-subject variability (BSV, or η) was ascribed to an exponential error model. Parameter shrinkage was calculated as [1 - sd(η)/ω], where sd(η) and ω are the standard deviation of individual η parameters and the population model estimate of the BSV, respectively. Standard errors were computed by stochastic approximation, not by linearization. The likelihood ratio test, including the log-likelihood, the Akaike information criterion (AIC) and the Bayesian information criterion (BIC), were used to test different hypotheses regarding the final model, the covariate effects on pharmacokinetic parameters, the residual variability model (proportional versus proportional plus additive error model) and the structure of the variance-covariance matrix for the BSV parameters. Residuals are presented as normalized prediction distribution errors (NPDEs), based on the estimates of unbiased means and variances of the predictions by using 500 Monte Carlo simulations of the final model. (The calculation includes a decorrelation step of the prediction errors.) The mean values and variance of these normalized residues must not be different from 0 and 1, respectively. Diagnostic graphics and other statistics were derived using the R software program [16]. The results are expressed as raw numbers (%), means ± SD or medians (ranges) for data with nonnormal distributions. *P *< 0.05 was considered significant.

## Results

**Figure 1:**
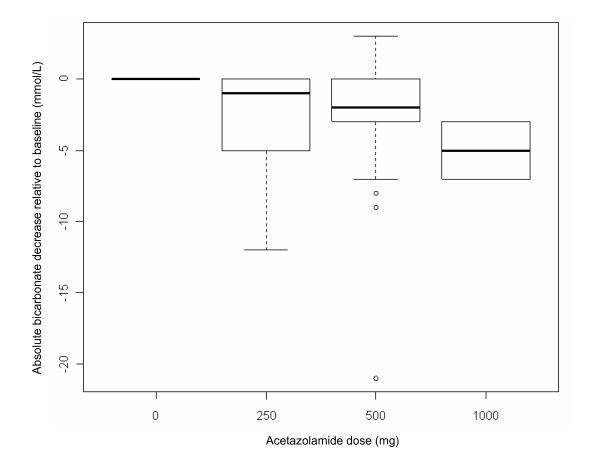
**Differences between pre-acetazolamide dose bicarbonate level and bicarbonate level at 24 hours in all patients, plotted according to the total dosage of acetazolamide administered**. Boxplots show the medians, first and third quartiles and 10th and 90th percentiles. The zero level at predose indicates that these values are based on intrapatient differences (repeated measures). Predose values: median 37.5; 25th and 75th percentiles 34 and 41, respectively; and 10th and 90th percentiles 30 and 45 mmol/L, respectively.

Of the 536 COPD patients identified, 446 patients received neither invasive mechanical ventilation nor ACET during their ICU stay. The files of 22 patients were incomplete and therefore were excluded. Sixty-eight patients were eligible for analysis. Patients' characteristics upon entry into the study are summarized in Table 1. None of the included patients received ventilation through a tracheostomy tube. Among the 68 investigated patients, 207 time-serum HCO_3_^- ^observations were available for analysis, with a median of three observations per patient (range, 1 to 6). Differences between the pre-ACET dose HCO_3_^- ^and HCO_3_^- ^levels at 24 hours in all patients, plotted according to the total dosage of ACET administered(Figure 1), showed a dose-response relationship. Additionally, the scale of observed differences was similar to what has previously been reported [9,10].

**Table 1:** Main characteristics of the study population (*N *= 68)^a^

Characteristics	Value
Age, years, median (range)	74 (44 to 99)
Male/female gender, *n *(%)	40 (58.9)/28 (41.1)
Smoker, pack-years, median (range)	50 (10 to 130)
Ex-smoker, *n *(%)	46 (67.6)
Diuretic treatment more than four weeks, *n *(%)	21 (30.9)
Systemic corticosteroid treatment more than four weeks, *n *(%)	5 (7.3)
Home oxygen therapy, *n *(%)	26 (38.2)
Home noninvasive ventilation, *n *(%)	6 (8.8)
Body weight, kg, median (range)	61.8 (34 to 142)
FEV_1_, mL, median (range)	800 (350 to 1940)
FEV_1_/FVC, %, median (range)	50.9 (24 to 66)
LVEF, %, median (range)	60 (20 to 80)
SAPS II at ICU admission, median (range)	47 (20 to 95)
Lenght of ICU stay, days, median (range)	21 (6 to 123)
Length of invasive mechanical ventilation, days, median (range)	18 (3 to 110)
ICU mortality, *n *(%)	16 (23.5)
Cause of respiratory failure	
Pneumonia, *n *(%)	22 (32.4)
Bronchitis, *n *(%)	17 (25)
Left ventricular failure, *n *(%)	5 (7.3)
Surgery, *n *(%)	4 (5.9)
Use of sedative drugs, *n *(%)	6 (8.8)
Pulmonary embolism, *n *(%)	1 (1.5)
Unknown, *n *(%)	13 (19.1)
Laboratory measurements before onset ACET administration	
pH, median (range)	7.45 (7.34 to 7.57)
PaCO_2_, mmHg, median (range)	55 (36 to 93)
PaO_2_, mmHg, median (range)	78 (41 to 136)
Serum bicarbonate, mmol/L, median (range)	37.5 (27 to 58)
Serum potassium, mmol/L, median (range)	3.8 (2.7 to 4.6)
Serum chloride, mmol/L, median (range)	96 (69 to 108)
Serum protein, g/L, median (range)	57 (40 to 72)
Serum creatinine, μmol/L, median (range)	64 (22 to 202)
Additional treatments during ACET administration	
Furosemide, *n *(%)	32 (47.1)
Systemic glucocorticoid, *n *(%)	10 (14.7)
β_2_-agonists, *n *(%)	8 (11.7)
Fluid load, mL, median (range)	1, 000 (0 to 2, 300)
Pressure-support ventilation, *n *(%)	31 (45.6)
Volume-assisted ventilation, *n *(%)	37 (54.4)

^a^ACET, acetazolamide; COPD, chronic obstructive pulmonary disease; FEV_1_, forced expiratory volume in one second; FVC, forced vital capacity; LVEF, left ventricular ejection fraction; PaCO_2_, partial pressure of carbon dioxide; PaO_2_, partial pressure of oxygen; SAPS II, Simplified Acute Physiology Score II.

### Pharmacodynamic modeling

**Figure 2:**
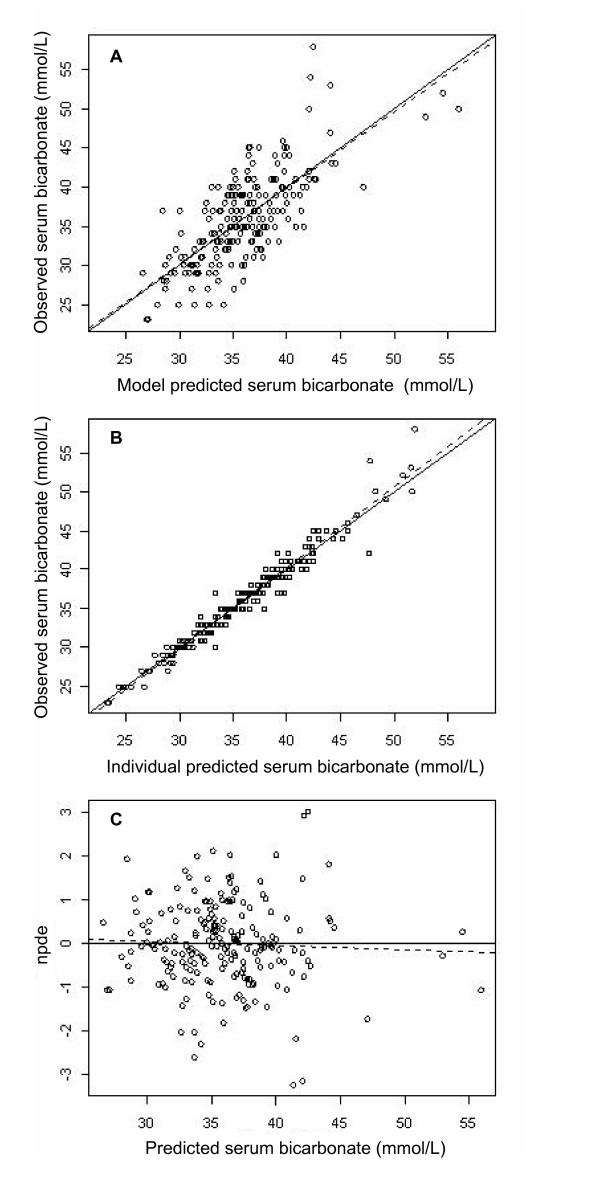
**Goodnees-of-fit plots for the final model of acetazolamide pharmacodynamics**. Shown are the results for 68 weaning chronic obstructive pulmonary disease patients. Observed versus model-predicted serum bicarbonate concentrations for **(A) **mean and **(B) **individual predictions and **(C) **normalized prediction distribution errors (NPDEs) versus predicted serum bicarbonate concentrations. The solid lines represent the identity lines and the dotted lines represent the regression lines. The mean and variance of the NPDE distribution were not significantly different from 0 and 1, respectively (*P *= 0.66 and *P *= 0.60, respectively; Wilcoxon signed-rank test and Fisher variance test, respectively) and from normality (*P *= 0.052, Shapiro-Wilks test), illustrating the robusteness of serum bicarbonate prediction after acetazolamide administration.

The turnover model satisfactorily described the data. The parameters of the model were Bicar_0 _(baseline), *k*_out _and A_50_. BSVs could be estimated for Bicar_0 _and *k*_out _only. It was not possible to simultaneously determine the E_max _and A_50 _parameters. We therefore decided to assign E_max _a value of 1. Residual variability was described by a proportional error model. Using the enteral route of ACET administration to predict serum HCO_3_^- ^concentration did not improve the model or the precision of the other parameters. Since the biodisponibility of ACET is excellent, the simplest pharmacokinetic (intravenous) model was therefore used for all patients. The main covariate effects on Bicar_0 _were SAPS II at ICU admission (*P *= 0.01), serum chloride concentration (*P *< 0.001) and concomitant glucocorticoid administration (*P *= 0.02). Coadministration of furosemide significantly decreased HCO_3_^- ^elimination (effect on *k*_out_). The final covariate submodel was then:

$$\text{Bica}{\text{r}}_{0}=\text{TV}\left(\text{Bica}{\text{r}}_{0}\right)\times {\left(\text{SAPS}\;\text{II}∕50\right)}^{-0.11}\times {\left(\text{chloride}∕100\right)}^{-1.17}\times \left(1.1\;\text{if}\;\text{glucocorticoids}\right)$$

and

$$k_{\text{out}}=\text{TV}\left(k_{\text{out}}\right)\times \left[1-\text{furosemie}\;\text{dosage}∕\left(\text{furosemide}\;\text{dosage}+\text{Fu}{\text{r}}_{50}\right)\right],$$

where TV is typical value and Fur_50 _denotes the dose of furosemide that induces a 50% decrease of *k*_out_. The AIC/BIC ratio was 1, 044/1, 066. When parameters were standardized to a SAPS II of 50 and a serum chloride level of 100 mmol/L, the dose of ACET provoking 50% of the putative maximal effect on serum HCO_3_^- ^(A_50_) was 117 +/- 21 mg. Additionally, we checked the likelihood profile of A_50_, confirming that the value of A_50 _was not 0 (data not shown). Table 2 summarizes the final population pharmacokinetic estimates. The predicted versus observed serum HCO_3_^- ^concentrations are depicted in Figures 2A, B and 2C, which show the corresponding normalized prediction distribution error tests for these data. The normalized prediction distribution error test plotted against the covariates of interest was also satisfactory, illustrating the robustness of the model (data not shown). A graph of the model errors over time was created by plotting measured model predicted values and measured individual predicted values over time for each subject. No systematic deviation of error was obvious over time (data not shown).

**Table 2:** Parameter estimates of the final acetazolamide population model in patients with chronic obstructive pulmonary disease (*N *= 68)^a^

Parameter	Estimate (%rse)	BSV (%rse) [shrinkage]
Half-life, day	0.25 (fixed)	NA
Bicar_0_, mmol/L	35.5 (2)	0.101 (9) [0.04]
× SAPS II effect (SAPS II/50)^-0.11^	-0.112 (39)	
× Corticosteroid effect, if present	1.092 (41)	
× Serum chloride effect (chloride/100) ^-1.17^	-1.18 (17)	
*k*_out_, mmol/day	0.395 (17)	0.792 (15) [0.27]
Fur_50_	187 (21)	
A_50_	117 (18)	NA
Residual variability	0.04 (8)	NA

^a^%rse, percentage relative standard error; ACET, acetazolamide; A_50_, ACET dosage that provokes 50% of putative maximal effect on serum bicarbonate; BSV, between-subject variability; Bicar_0_, bicarbonate baseline level; Fur_50_: furosemide dose that induces a 50% decrease of *k*_out_; *k*_out_, first-order constant rate of bicarbonate elimination; NA, not applicable; SAPS II, Simplified Acute Physiology Score II; TV: typical value. When COPD patients received ACET, the effect of covariates on observed serum bicarbonate levels was Bicar_0 _= TV(Bicar_0_) × (SAPS II/50)^-0.11 ^× (chloride/100)^-1.17 ^× (1.1 if glucocorticoids) and *k*_out _= TV(*k*_out_) × [1 - furosemide dosage/(furosemide dosage + Fur_50_)]. Shrinkage was calculated as [1 - sd(η)/ω], where sd(η) and ω are the standard deviation of individual η parameters and the population model estimate of the BSV, respectively.

### ACET dosing in COPD patients

**Figure 3:**
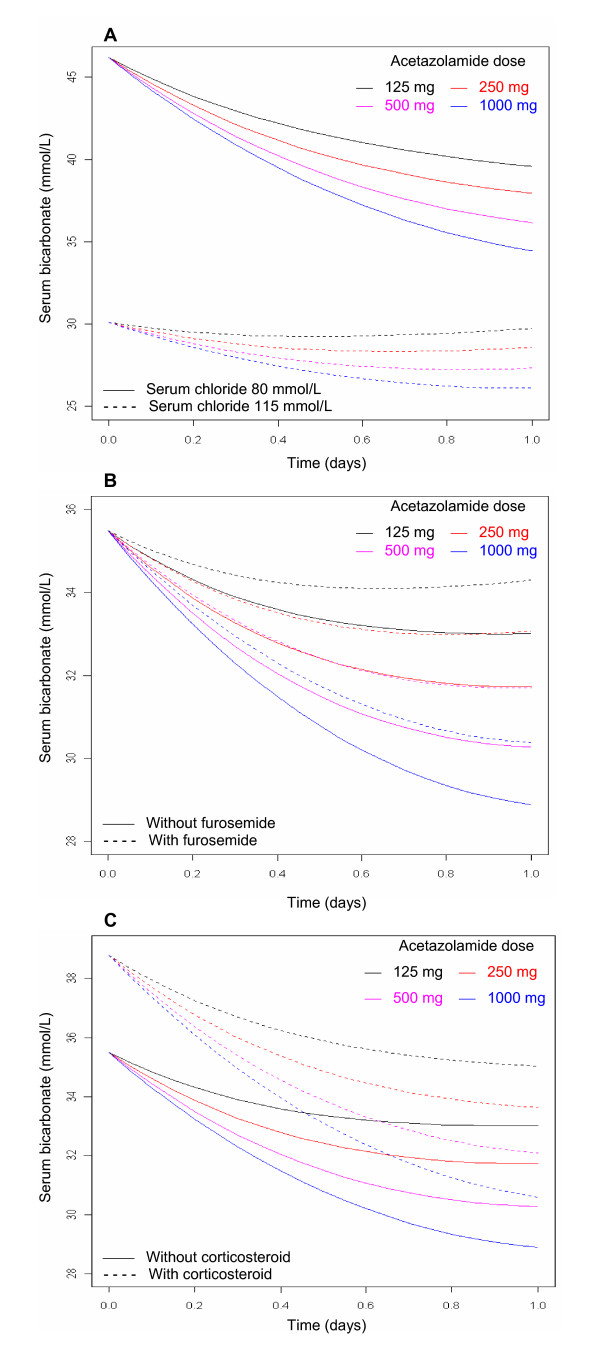
**Model-predicted effect of 125 mg, 250 mg, 500 mg or 1, 000 mg of acetazolamide**. Shown are the effects of acetazolamide (ACET) administered once daily on serum bicarbonate in the presence of **(A) **low or high levels of serum chloride, **(B) **coprescription or not of 20 to 160 mg/day furosemide or **(C) **coprescription or no coprescription of 1 mg/kg/day systemic cortocosteroids. Modelization of ACET pharmacodynamics was derived from 68 chronic obstructive pulmonary disease patients during the weaning period with metabolic alkalosis (Simplified Acute Physiology Score II at ICU admission was standardized at 50 and is shown in all parts of the figure). A higher ACET dosage is required to reduce serum bicarbonate concentrations > 5 mmol/L when high chloride or coprescription of systemic corticosteroids or furosemide occurs.

Using this pharmacodynamic model, we assessed the effect of ACET administered once daily on serum HCO_3_^- ^levels under various conditions of serum chloride level and coprescription of furosemide or glucocorticoids (Figures 3A, B and 3C). According to our model, a higher ACET dosage is required to reduce serum HCO_3_^- ^concentration > 5 mmol/L in the presence of high levels of serum chloride or coprescription of systemic corticosteroids or furosemide.

## Discussion

Little is known about the pharmacokinetics or pharmacodynamics of ACET in acutely ill patients, especially COPD patients during the weaning process [17]. At low doses (4 mg/kg), ACET preferentially and fully inhibits renal and endothelial cell CA [18,19], whereas at higher doses (7 to 12 mg/kg), ACET inhibits the activity of CA in red blood cells, chemoreceptors and respiratory muscles [19]. These findings underline the complexity of the response to ACET [8,20]. Morover, ACET administration is associated with almost no severe side effects. ACET administered at the usual dosage to COPD patients during the weaning process is known to induce a modest but significant decrease in serum HCO_3_^- ^concentration [10]. Higher doses of the drug could therefore be given to mechanically ventilated COPD patients to improve clinical respiratory variables. Our study is the first, to the best of our knowledge, to use population pharmacodynamic modeling to characterize the dose-response relationship associated with ACET response in invasively ventilated COPD patients. None of the patients included in this study experienced undesirable side effects after ACET administration, confirming the relative safety of the drug.

ACET is commonly used to treat metabolic alkalosis. ACET inhibits the CA enzyme and induces metabolic acidosis [8]. Metabolic acidosis in turn induces an increase in both minute ventilation and PaO_2_, potentially allowing COPD patients to be weaned more rapidly from mechanical ventilation [6,7]. We therefore decided to use serum HCO_3_^- ^concentration as a substitute for the effect of ACET, taking into account parameters that might influence HCO_3_^- ^concentration over time.

In our model, the E_max _parameter could not be estimated, probably because of a lack of data with high enough concentrations. With a 117-mg dose of ACET that produces a putative half-maximal effect, this model predicts that a dosage of ACET > 500 mg twice daily could decrease serum HCO_3_^- ^concentration > 5 mmol/L in the presence of high serum chloride concentration or coadministration of furosemide or corticosteroids. The main covariates that had an effect on ACET pharmacodynamics were SAPS II at ICU admission, serum concentration of chloride and the coadministration of corticosteroids or furosemide. The dosage based on these parameters is expected to improve the benefits of ACET in COPD patients with metabolic alkalosis.

We showed that few covariates affected ACET pharmacodynamics in mechanically ventilated COPD patients. Both furosemide and corticosteroids induce metabolic alkalosis by stimulation (by different mechanisms) of distal tubular H^+ ^secretion [21]. Higher doses of ACET may be needed to counterbalance the effect of these drugs. Additionally, ACET and furosemide are transported by the same carrier-mediated mechanism from plasma to their site of action localized at the luminal side of renal tubules [22]. Furosemide may therefore decrease the cotransport of ACET by a competitive mechanism. Our results are of import, since many COPD patients are treated by corticosteroids or furosemide. Indeed, half the population in our study received furosemide during the weaning period. Chloride and HCO_3_^- ^are the two major anions in the serum. Physicochemical principles of conservation of mass and electroneutrality imply that a high serum chloride concentration is likely to be associated with a relatively low HCO_3_^- ^concentration. In the presence of a higher chloride concentration (and thus a lower HCO_3_^- ^concentration), non-CA-dependent mechanisms of HCO_3_^- ^reabsorption becomes activated and contributes to a greater extent in HCO_3_^- ^reclamation when the total filtered HCO_3_^- ^falls [23-25].

The role of SAPS II on ACET pharmacodynamics remains speculative. The SAPS II includes the serum level of HCO_3_^- ^at ICU admission. We hypothesize that the most severely ill COPD patients at ICU admission may have a higher respiratory acidotic component of alkalosis at readiness to wean, limiting the effect of 500 mg of ACET [10].

The single biggest factor affecting ACET elimination is renal clearance. In our model, serum creatinine was not a significant covariate, probably because none of our patients had severe renal impairment. However, renal function is embedded in the SAPS II, which is a covariate of interest in our model. Additionally, the ACET dosage derived from our model are only valid if an elimination half-life of ACET of six hours is assumed. Our model therefore cannot be used to describe the pharmacodynamics of ACET in patients with kidney failure. Lastly, the volume of distribution of HCO_3_^- ^is approximately that of total body water. Body weight as a substitute for total body water was not found to affect the response to ACET in our model.

In a recent case-control study, we showed that ACET given at the dosage of 500 mg/day significantly but moderately diminished serum HCO_3_^- ^without otherwise effecting arterial blood gases (except for the PaO_2_/fraction of inspired O_2 _ratio) or respiratory parameters in weaning COPD patients [10]. Taken together, these findings indicate that, during the weaning period, COPD patients could benefit from a higher ACET dosage for effective reversal of metabolic alkalosis.

We show here that in mechanically ventilated COPD patients there is a dose-response relationship after administration of ACET to decrease serum HCO_3_^-^, with a putative half-maximal effective dose of 117 mg. Berthelsen and colleagues [17], in a study of heavily sedated alkalotic patients, showed that increasing doses of ACET were associated with increased excretion of urinary HCO_3_^- ^over a short period of time. Our results differ from those reported in several previous studies. Mazur and colleagues [9] showed that ACET administered as a single 500-mg dose reversed metabolic alkalosis in mechanically ventilated patients as effectively as four administrations of 250 mg, suggesting that there is no dose-response relationship when using ACET. Similarly, we did not find a relationship between preexisting respiratory function (including baseline forced expiratory volume in one second) and response to ACET administration in acutelly ill COPD patients, contrary to previously published data derived from more stable COPD patients [8,26]. The discrepancy between our results and those reported in previous studies are likely due to differences in illness severity at admission, serum chloride levels at admission and coprescription of corticosteroids or furosemide.

One of the limitations of this study is that patients at a single center were included. Our patient population was nonetheless comparable to similar COPD patients treated with invasive mechanical ventilation [27]. Another possible limitation is that some patients presented compensated respiratory acidosis without real overlapping metabolic alkalosis, reflecting the variety of acid-base disorders present in mechanically ventilated COPD patients [10]. To the best of our knowledge, the pH range observed in the present study has not yet been reported to influence CA inhibition by ACET.

## Conclusions

In conclusion, an increased ACET dosage could be beneficial in the treatment of COPD patients receiving corticosteroids or furosemide or presenting with high serum chloride concentrations. These assumptions should be prospectively confirmed, since our conclusions are drawn on the basis of studying only 68 patients. Unanswered questions remain, such as the effect of ACET administration on PaCO_2 _levels and minute ventilation in these patients. Finally, the effect of ACET administration on COPD patients in terms of mortality and length of mechanical ventilation remains to be determined.

## Key messages

• Little is known about the pharmacodynamics of ACET in the critically ill.

• We used a population pharmacodynamic model to characterize the dose-response relationship associated with ACET response in invasively ventilated COPD patients.

• According to our model, an ACET dosage > 500 mg twice daily is required to reduce serum HCO_3_^- ^concentrations by > 5 mmol/L in the presence of high serum chloride concentrations or coadministration of furosemide or corticosteroids.

## Abbreviations

%rse: percentage relative standard error; A_50_: ACET dosage that induces 50% of the maximal effect; ACET: acetazolamide; AIC: Akaike information criterion; A(t): ACET amount in the body; BIC: Bayesian information criterion; Bicar(t): serum bicarbonate level; Bicar_0_, bicarbonate baseline level; BSV: between-subject variability; CA: carbonic anhydrase; COPD: chronic obstructive pulmonary disease; E_ACET_: ACET effect; E_max_: maximal effect of ACET; FEV_1_: forced expiratory volume in one second; Fur_50_: dose of furosemide that induces a 50% decrease of *k*_out_; FVC: forced vital capacity; G(t): ACET amount in gut; HCO_3_^-^: bicarbonate; *k*_a_: first-order absorption rate; *k*_elim_: constant rate of elimination; *k*_in_: bicarbonate rate formation; *k*_out_: first-order constant rate of bicarbonate elimination; LVEF: left ventricular ejection fraction; NA, not applicable; SAPS II: Simplified Acute Physiology Score II; TV: typical value.

## Competing interests

The authors declare that they have no competing interests.

## Authors' contributions

NH collected data and drafted the manuscript. CF conceived of the study, participated in its design and coordinated and drafted the manuscript. SU analyzed the data and drafted the manuscript. All authors read and approved the final manuscript.

## References

[B1] Lopez AD Shibuya K Rao C Mathers CD Hansell AL Held LS Schmid V Buist S Chronic obstructive pulmonary disease: current burden and future projections Eur Respir J 2006 27 397 412 10.1183/09031936.06.00025805 16452599

[B2] Gunen H Hacievliyagil SS Kosar F Mutlu LC Gulbas G Pehlivan E Sahin I Kizkin O Factors affecting survival of hospitalised patients with COPD Eur Respir J 2005 26 234 241 10.1183/09031936.05.00024804 16055870

[B3] Lightowler JV Wedzicha JA Elliott MW Ram FS Non-invasive positive pressure ventilation to treat respiratory failure resulting from exacerbations of chronic obstructive pulmonary disease: Cochrane systematic review and meta-analysis BMJ 2003 326 185 10.1136/bmj.326.7382.185 12543832 PMC140272

[B4] Gursel G Determinants of the length of mechanical ventilation in patients with COPD in the intensive care unit Respiration 2005 72 61 67 10.1159/000083402 15753636

[B5] Khanna A Kurtzman NA Metabolic alkalosis Respir Care 2001 46 354 365 11262555

[B6] Berthelsen P Gøthgen I Husum B Jacobsen E Oxygen uptake and carbon dioxide elimination after acetazolamide in the critically ill Intensive Care Med 1985 11 26 29 3918091 10.1007/BF00256061

[B7] Brimioulle S Berre J Dufaye P Vincent JL Degaute JP Kahn RJ Hydrochloric acid infusion for treatment of metabolic alkalosis associated with respiratory acidosis Crit Care Med 1989 17 232 236 10.1097/00003246-198903000-00006 2493354

[B8] Swenson ER Carbonic anhydrase inhibitors and ventilation: a complex interplay of stimulation and suppression Eur Respir J 1998 12 1242 1247 10.1183/09031936.98.12061242 9877470

[B9] Mazur JE Devlin JW Peters MJ Jankowski MA Iannuzzi MC Zarowitz BJ Single versus multiple doses of acetazolamide for metabolic alkalosis in critically ill medical patients: a randomized, double-blind trial Crit Care Med 1999 27 1257 1261 10.1097/00003246-199907000-00004 10446816

[B10] Faisy C Mokline A Sanchez O Tadié JM Fagon JY Effectiveness of acetazolamide for reversal of metabolic alkalosis in weaning COPD patients from mechanical ventilation Intensive Care Med 2010 36 859 863 10.1007/s00134-010-1795-7 20217045

[B11] Rabe KF Hurd S Anzueto A Barnes PJ Buist SA Calverley P Fukuchi Y Jenkins C Rodriguez-Roisin R van Weel C Zielinski J Global Initiative for Chronic Obstructive Pulmonary Disease Global strategy for the diagnosis, management, and prevention of chronic obstructive pulmonary disease: GOLD executive summary Am J Respir Crit Care Med 2007 176 532 555 10.1164/rccm.200703-456SO 17507545

[B12] Boles JM Bion J Connors A Herridge M Marsh B Melot C Pearl R Silverman H Stanchina M Vieillard-Baron A Welte T Weaning from mechanical ventilation Eur Respir J 2007 29 1033 1056 10.1183/09031936.00010206 17470624

[B13] Le Gall JR Lemeshow S Saulnier F A new Simplified Acute Physiology Score (SAPS II) based on a European/North American multicenter study JAMA 1993 270 2957 2963 10.1001/jama.270.24.2957 8254858

[B14] Yano I Takayama A Takano M Inatani M Tanihara H Ogura Y Honda Y Inui K Pharmacokinetics and pharmacodynamics of acetazolamide in patients with transient intraocular pressure elevation Eur J Clin Pharmacol 1998 54 63 68 10.1007/s002280050422 9591933

[B15] Ritschel WA Paulos C Arancibia A Agrawal MA Wetzelsberger KM Lücker PW Pharmacokinetics of acetazolamide in healthy volunteers after short- and long-term exposure to high altitude J Clin Pharmacol 1998 38 533 539 9650543 10.1002/j.1552-4604.1998.tb05791.x

[B16] Ihaka R Gentleman R R: a language for data analysis and graphics J Comput Graphic Stat 1996 5 299 314 10.2307/1390807

[B17] Berthelsen P Gøthgen I Husum B Jacobsen E Dissociation of renal and respiratory effects of acetazolamide in the critically ill Br J Anaesth 1986 58 512 516 10.1093/bja/58.5.512 3083849

[B18] Teppema L Dahan A Acetazolamide and breathing: Does a clinical dose alter peripheral and central CO_2 _sensitivity? Am J Resp Crit Care Med 1999 160 1592 1597 10556126 10.1164/ajrccm.160.5.9903088

[B19] Kiwull-Schöne HF Teppema LJ Kiwull PJ Low-dose acetazolamide does affect respiratory muscle function in spontaneously breathing anesthetized rabbits Am J Respir Crit Care Med 2001 163 478 483 11179126 10.1164/ajrccm.163.2.9911075

[B20] Wagenaar M Teppema L Berkenbosch A Olievier C Folgering H The effect of low-dose acetazolamide on the ventilatory CO_2 _response curve in the anaesthetized cat J Physiol 1996 495 227 237 8866365 10.1113/jphysiol.1996.sp021587 PMC1160738

[B21] Webster NR Kulkarni V Metabolic alkalosis in the critically ill Crit Rev Clin Lab Sci 1999 36 497 510 10.1080/10408369991239286 10560889

[B22] Uway Y Saito H Hashimoto Y Inui KI Interaction and transport of thiazide diuretics, loop diuretics, and acetazolamide via rat renal organic anion transporter rOAT1 J Pharmacol Exp Ther 2000 295 261 265 10991988

[B23] Maren TH Chemistry of the renal reabsorption of bicarbonate Can J Physiol Pharmacol 1974 52 1041 1050 10.1139/y74-138 4217649

[B24] Maren TH Carbonic anhydrase: chemistry, physiology, and inhibition Physiol Rev 1967 47 595 781 4964060 10.1152/physrev.1967.47.4.595

[B25] Preisig PA Toto RD Alpern RJ Carbonic anhydrase inhibitors Ren Physiol 1987 10 136 159 3133728 10.1159/000173126

[B26] Skatrud JB Dempsey JA Relative effectiveness of acetazolamide versus medroxyprogesterone acetate in correction of chronic carbon dioxide retention Am Rev Respir Dis 1983 127 405 412 6404203 10.1164/arrd.1983.127.4.405

[B27] Groenewegen KH Scholas AM Wouters EF Mortality and mortality-related factors after hospitalization for acute exacerbation of COPD Chest 2003 124 459 467 10.1378/chest.124.2.459 12907529

